# Complete Genome Sequence of a Field Isolate of Classical Swine Fever Virus Belonging to Subgenotype 2.1b from Hunan Province, China

**DOI:** 10.1128/genomeA.00763-15

**Published:** 2015-07-23

**Authors:** Weixing Shao, Shuang Liu, Faxing Wu, Zhi Zhang, Yaqin Dong, Xiaocheng Li

**Affiliations:** Division of Surveillance on Livestock Disease, China Animal Health and Epidemiology Center, Qingdao, China

## Abstract

We report the complete genome sequence of a field isolate of classical swine fever virus (CSFV), Hunan 23/2013, belonging to the predominant subgenotype 2.1b. This strain was originally isolated from diseased pigs in Hunan Province, China. This report will help in understanding the molecular diversity of CSFV stains circulating in China and in selecting and developing a suitable vaccine candidate for CSF control.

## GENOME ANNOUNCEMENT

Classical swine fever (CSF) is a highly contagious, fatal disease of pigs and wild boars caused by the CSF virus (CSFV), a positive-sense RNA virus belonging to the genus *Pestivirus* within the family *Flaviviridae* (1). The CSFV genome is approximately 12.3 kb in length and encodes a single polyprotein that is co- and post-translationally cleaved to form mature structural and nonstructural proteins (2, 3). Phylogenetic analysis based on sequenced data sets of the E2 envelope glycoprotein gene divides CSFVs into three genotypes (I, II, III), with each being further divided into three or four subgenotypes (1.1 to 1.3, 2.1 to 2.3, and 3.1 to 3.4) (4). CSFV subgenotype 2.1 has been further divided into 2.1a and 2.1b (5, 6). In mainland China, CSFVs of subgenotype 2.1b have predominated since the 1990s (7–9).

Here, we describe the complete genome sequence of a field isolate of CSFV, GXF29/2013, which was isolated from the mixture of spleen and lymph nodes of a suspected case from an intensive pig farm in Hunan Province, China, in 2013. The full nucleotide sequence was obtained from four overlapping fragments amplified by reverse transcription-PCR (RT-PCR). Total RNA was isolated using Trizol reagent, and cDNA was synthesized with AccuScript High-Fidelity reverse transcriptase using CSFV-specific oligonucleotide primers. The overlapping genome fragments were amplified from cDNA, and four amplified PCR products were purified and cloned into vector PMD-19. The recombinant plasmids were sequenced. Sequences were assembled and manually edited to form the final genome sequence.

The complete genome sequence of the isolate Hunan 23/2013 is 12,295 nucleotides (nt) in length, with a 5′ untranslated region (UTR) of 372 nt and a 3′ UTR OF 226 nt. A single large open reading frame (ORF) is 11,697 nt between nt positions 373 and 12,069 and is capable of coding for a polyprotein of 3,898 amino acids.

Based on phylogenetic analysis of the E2 fragment of Hunan 23/2013 and compared with 28 reference strains—except CSF0705 (Cuba), CSF1057 (Cuba), and GXGL-1/06 (10)—Hunan 23/2013 was located into subgenotype 2.1b. The difference between the sequences of the E2 fragment of Hunan 23/2013 and those of the subgenotype 2.1b CSFVs was 3.2 to 5.6%. However, more significant levels of nucleotide difference were found between the E2 fragment of Hunan 23/2013 and those of the subgenotype 2.1a, 2.1b, 1.1, 2.2, and 2.3 CSFVs: 7.4 to 7.9%, 11.7 to 12.4%, 19.4 to 20.9%, 13.1 to 14.4%, and 16.7 to 19.5%, respectively.

Although CSFV subgenotype 2.1b is predominant in China, this is the first report of a complete genome sequence (12,295 bp) of a CSFV field isolate from a suspected case of CSF in China that belongs to subgenotype 2. The information about the complete genome of the Hunan 23/2013 isolate in the present study will enrich the data of CSFV in GenBank and help in understanding the molecular diversity of CSFVs circulating worldwide, which may have implications for the prevention and control of CSFV infection in pigs in China.

### Nucleotide sequence accession number.

The complete genome sequence of CSFV Hunan 23/2013 has been deposited in GenBank under the accession number KP233071.
